# Evolution of the Corrosion Product Film on Nickel-Aluminum Bronze and Its Corrosion Behavior in 3.5 wt % NaCl Solution

**DOI:** 10.3390/ma12020209

**Published:** 2019-01-09

**Authors:** Yang Ding, Rong Zhao, Zhenbo Qin, Zhong Wu, Liqiang Wang, Lei Liu, Weijie Lu

**Affiliations:** 1 State Key Laboratory of Metal Matrix Composites, Shanghai Jiao Tong University, Shanghai 200240, China; dyjames@sjtu.edu.cn (Y.D.); cm329ing@gmail.com (R.Z.); wang-liqiang@sjtu.edu.cn (L.W.); 2 Collaborative Innovation Center for Advanced Ship and Deep-Sea Exploration, Shanghai 200240, China; 3 School of Material Science and Engineering, Tianjin University, Tianjin 300072, China; zhenboqin89@163.com (Z.Q.); wuzhong2319@163.com (Z.W.)

**Keywords:** nickel-aluminum bronze, corrosion product film, in-situ SVET, in-situ AFM

## Abstract

The in-situ studies of the corrosion product film on nickel-aluminum bronze are significant for explaining the mechanism of its corrosion resistance. In this paper, the corrosion behavior of nickel-aluminum bronze and the formation process of the protective film in 3.5 wt % NaCl solution are systematically investigated. The results of scanning electron microscope analysis and electrochemical tests indicate that the corrosion resistance of nickel-aluminum bronze is improved due to the formation of the corrosion product film. The change of local electrochemical property on the corrosion product film during the immersion time is evaluated via in-situ scanning vibrating electrode technique, and it reveals the evolution rules of ionic flux in real time. The formation process of the protective film on different phases in nickel-aluminum bronze is observed directly by in-situ atomic force microscopy as height change measurements. The α phases at different locations present different corrosion behaviors, and the lamellar α phase within the α + κ_III_ eutectoid structure gets more serious corrosion attack. The κ phases establish a stable and dense protective film in short time, preventing the corrosion attack effectively. The β′ phase, however, suffers the most serious corrosion damage until a protective film is formed after 150 min of immersion.

## 1. Introduction

Nickel-aluminum bronze (NAB) alloy represents a branch of Cu–Al-based alloy containing about 4–5 wt % Ni, 8.5–9.5 wt % Al, 3.5–4.5 wt % Fe, and 0.8–1.5 wt % Mn. As a multicomponent alloy, NAB alloy consists of copper-rich coarse α phase, retained martensitic β phase (β′), and four kinds of aluminum-rich κ phases which are distinguished by their morphology, composition, and distribution [1,2,3,4]. Both κ_I_ and κ_II_ are Fe_3_Al-based intermetallic precipitates with globular or rosette shape. Moreover, κ_I_ precipitates are larger in size and rarely formed in NAB alloys when Fe content is less than 5 wt % [3]; κ_II_ phases are unevenly distributed at the boundaries of α and β′ phases and are about 1–5 μm in diameter [5]. NiAl-based κ_III_ phases are lamellar or rod-like (degenerate lamellar) structure eutectoid product with α phases near the α/β′ boundaries. Iron-rich κ_IV_ phases are fine particle precipitates distributed throughout α grains and leave a precipitate-free zone at the edge of α grains [6,7].

NAB alloy has been widely used for marine engineering equipment, by virtue of its good combination of mechanical properties and corrosion resistance [8,9,10]. Considerable research has been devoted to studying the corrosion behavior of NAB alloy to try and reveal the mechanism of corrosion attack. Song et al. [11] tested the corrosion properties of as-casted and friction-stir processed (FSPed) NAB. They reported that the corrosion behavior was sensitive to the microstructures and phases in NAB alloy, and the selective phase corrosion was a common phenomenon in immersion. It was found that the lamellar α + κ_III_ eutectoid structures and β′ phases were readily corroded in neutral sodium chloride solution. Additionally, it is well accepted that the corrosion resistance of NAB alloy was from a sustainable and strongly adherent corrosion product film on fresh alloy, which could prevent the alloy from being corroded further [12,13,14,15]. Therefore, the study of corrosion product film on NAB alloy has also attracted the attention of many researchers. Schüssler et al. [14] investigated the structure and the passivation of the protective film on as-casted NAB by Auger electron spectroscopy (AES) and polarization curves. The results showed that the protective film mainly consisted of cuprous oxide in the outer layer and aluminum-oxide in the inner layer. Moreover, they declared that the outer cuprous oxide layer was responsible for reducing the charge transfer rate of the cathodic oxygen reduction, and the inner aluminum oxide layer hampered the ionic transport across the corrosion product by the anodic passivation. Using a crevice corrosion test on NAB alloy, Wharton et al. [16] found that the value of pH affected the formation of oxide protective film. The aluminum oxide film on κ phases is dissolved in acid solution, resulting in the fact that the κ phases act anodic to α phase matrix, and show a sharp corrosion attack. Qin et al. [17] reported a novel nickel ion implantation technology, which effectively improved the corrosion resistance of NAB alloy by forming the more compact protective film. The typical selective phase corrosion in NAB alloy gave way to turn into the uniform corrosion by nickel ion implantation technology.

Most existing research mainly focuses on investigating the composition, structure, and macroscopic corrosion properties of corrosion product film on NAB. However, the details of the film morphology evolution on various phases and the changes of electrochemical properties during the formation process are usually neglected. Besides, considering the complex application condition, the protective film formed on NAB alloy was usually damaged by high velocities, cavitation, or particle impingement, and so the formation of corrosion product film occurs throughout the whole service life of NAB alloy [13]. Therefore, to gain comprehensive insight into the formation of corrosion product film and provide a better strategy for improvement of corrosion resistance, further efforts are required to reveal the evolution process and anti-corrosion mechanism of corrosion product film on NAB alloy. 

Based on these considerations, scanning electron microscope (SEM) and electrochemical performance measurements are adopted to characterize the macroscopic corrosion behavior during the film formation process. In-situ scanning vibrating electrode technique (SEVT) and in-situ atomic force microscope (AFM) analytical techniques are conducted to reveal the changes of local electrochemical properties and height morphology. These in-situ measurements can present a direct picture of the practical corrosion and film formation process of NAB alloys in real time.

## 2. Experimental Procedures

### 2.1. Materials

**Figure 1:**
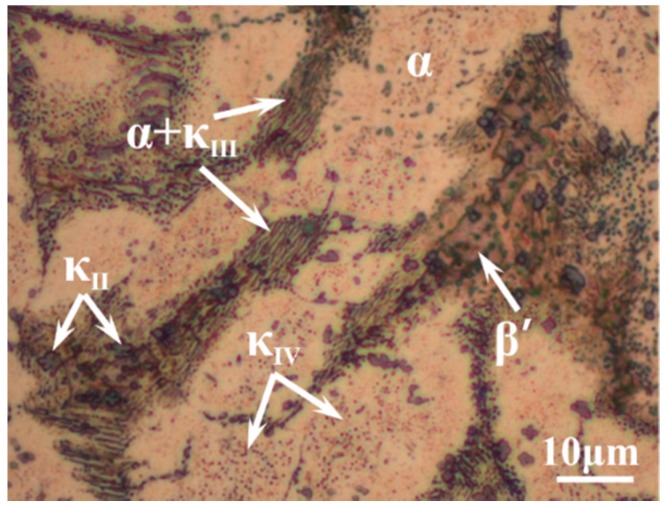
Optical micrograph of the microstructure of NAB alloy.

According to the chemical composition of C95800 in ASTM B148 standard, the casting materials, like pure copper, nickel, iron, manganese, and copper–aluminum alloy, were placed in a crucible of vacuum induction furnace together. After smelting at 1250 °C for 40 min in vacuum conditions, the molten metal was poured into a sand mold, and then a NAB alloy cast ingot with a dimension of φ 150 mm × 200 mm was prepared. All the specimens used in this work were sectioned from the same position of the cast ingot with a dimension of 10 mm × 10 mm × 5 mm to avoid composition variations. The chemical composition of NAB was examined by X-ray fluorescence spectrometer (Shimadzu, Kyoto, Japan) as 9.22 wt % Al, 4.17 wt % Fe, 4.17 wt % Ni, 1.23 wt % Mn, and Cu as balance. The microstructure of NAB alloy was shown in Figure 1. The α phase (white area) is a type of Cu-rich solid solution with face-centered cubic structure, and it is the main matrix in NAB alloy. The β′ phase, located between the α phase grains, is a metastable solid solution with martensitic structure at room temperature. The κ_II_ is a Fe_3_Al-based intermetallic precipitate with globular or rosette shape, while κ_IV_ is an iron-rich phase with fine particle shape that occurred within the α grains. The κ_III_ is a kind of NiAl-based lamellar or rod-like structure eutectoid product with α phases. Table 1 shows the chemical compositions of each phase in the NAB alloy. The composition of the κ_IV_ phase is not included, since its size is too small to be detected accurately. 

All the NAB specimens for immersion tests and microstructure observations were ground with SiC paper up to 2000 grit, polished with diamond paste to 0.5 μm, and then degreased in acetone and blow-dried. The microstructures were observed by an Axiocam MRc5 optical microscope (OM) (Zeiss, Heidenheim, Germany) after chemical etching by a solution consisting of 5 g FeCl_3_ + 2 mL HCl + 95 mL C_2_H_5_OH, for 10 s.

### 2.2. Experimental Methods

Immersion tests were performed in aerated neutral 3.5 wt % NaCl solution at an environmental temperature of 20 ± 2 °C for 48, 120, and 240 h. The morphology of corrosion product film on specimen surface was observed on an S-4800 field emission gun SEM (FEI, Hillsboro, OR, USA) equipped with energy dispersive X-ray spectroscopy (EDS) analyzer (Oxford Instruments, Oxford, UK). The electrochemical corrosion properties of the protective films after various immersion times were characterized on a CHI 660E electrochemical system using a conventional three-electrode cell with a saturated calomel reference electrode, a specimen working electrode, and a platinum foil counter electrode. The specimens, as the working electrode, had an exposed area of 1 cm^2^ to the electrolyte. The potentiodynamic polarization (PDP) sweep was conducted at a rate of 0.5 mV/s to obtain the anodic and cathodic Tafel slopes. The electrochemical impedance spectroscopy (EIS) experiment was carried out at a steady open circuit potential (E_ocp_) in the frequency range from 100 kHz to 0.01 Hz, with an alternating current amplitude of 5 mV. Each electrochemical measurement was repeated at least three times for reproducibility.

Scanning vibrating electron technique (SVET) measurement was introduced to investigate the in-situ change of current density distribution during the protective film formation process. The observation field was set on the NAB specimen after 240 h immersion in 3.5 wt % NaCl solution, with a 1.5 mm diameter artificial scratch circular region without corrosion product film, where the fresh metal substrate was exposed. Current density distribution above the specific specimen surface was examined in-situ, in 3.5 wt % NaCl solution, on an Applicable Electronics Inc. commercial SVET system with an ASET control program (Sciencewares, Falmouth, MA, USA) and a Pt–Ir vibrating electrode probe (MicroProbes Inc., San Jose, CA, USA). The Pt–Ir electrode probe tip was a platinum black sphere with 20 μm diameter. The distance of electrode probe tip to the specimen surface was kept at 100 μm, and the vibration frequency in the perpendicular direction to the surface was 88 Hz, with an amplitude of 30 μm. The time of acquisition for each SVET data point required 0.8 s, including 0.5 s stable testing time and 0.3 s moving time. The current density results of SVET were calibrated by deducting the background value, which was the average value of current densities obtained before and after the test in the solution far away from the specimen. 

The in-situ AFM with high resolution topography measurement was chosen to display the nanoscale topography changes of the protective film on the various phases during the formation process, which is complementary to the SVET measurement. The in-situ AFM measurement was conducted on a Dimension Icon & FastScan Bio AFM system (Bruker, Billerica, MA, USA) in contact mode at open circuit potential, with a scan rate of 0.5 Hz. The sharp nitride lever probe was a Bruker^TM^ SNL-10 D (Bruker, Billerica, MA, USA) with a nominal tip radius of 2 nm, nominal resonance frequencies of 18 kHz, and nominal spring constant of 0.06 N/m. Before scanning, the specimen was vibration polished for 2 h, and checked on the OM to determine the observation field. In the scanning interval, the specimen was immersed in the bottom of a glass petri dish containing 3.5 wt % NaCl solution, and then it was rinsed with acetone before drying.

## 3. Results and Discussion

### 3.1. Morphology Observation

**Figure 2:**
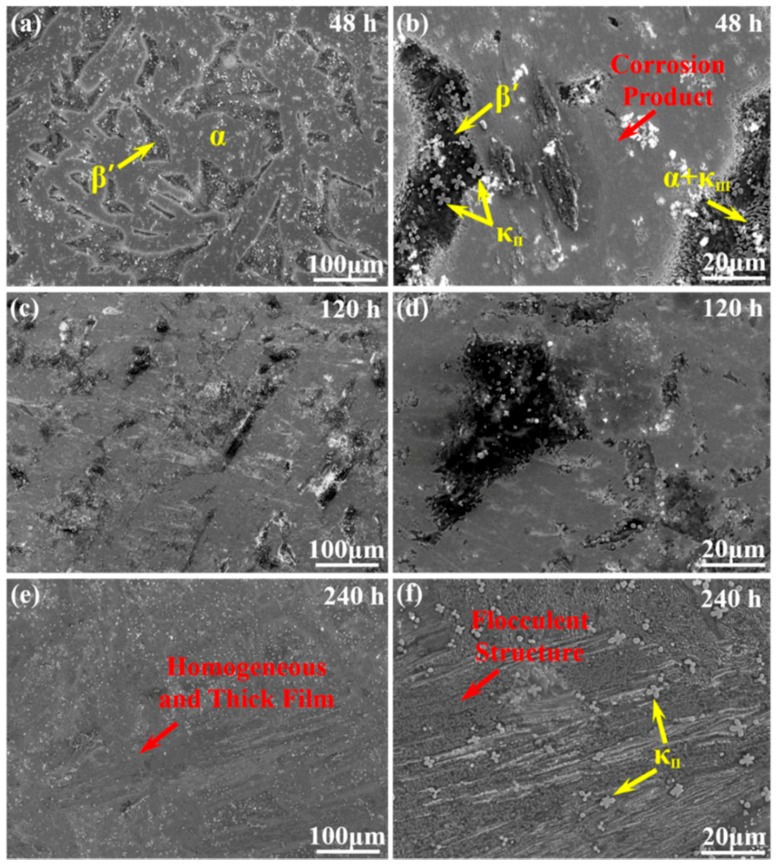
Morphology of corrosion product film on the surface of NAB alloy after immersion in 3.5 wt % NaCl solution for varying periods of time: (**a**,**b**) 48 h; (**c**,**d**) 120 h; (**e**,**f**) 240 h.

Figure 2 presents the morphology of corrosion product film on NAB alloy after different immersion times. After 48 h of immersion in NaCl solution, the oxide film on the various phases present a substantial difference, as shown in Figure 2a,b. The matrix α phases start to be covered by a thin and homogeneous corrosion product film, separating the internal fresh metal from corrosive medium. By contrast, the β′ phases, as well as the α + κ_III_ eutectoid structure, are visibly attacked with no protective film, leaving an obvious selective corrosion area. Moreover, the κ_II_ phases located in β′ phases are reserved completely, which are clearly observed in the magnification imaging. This phenomenon could be related to the cathodic behavior of κ phases as a very thin corrosion film formed on them in neutral 3.5 wt % NaCl solution [12]. With the increase of the immersion time (up to 120 h), the bare surface area of β′ phases and the α + κ_III_ eutectoid structure is reduced because of the deposition of corrosion product (Figure 2c,d). Clearly, the previous film covering on the α phases becomes thicker, and most of the phases are covered up. After 240 h of immersion, the entire surface of the NAB alloy is covered with the thick and homogeneous corrosive product film, as shown in Figure 2e. Different from the previous ones, the discontinuous flocculent structures are easily observed on the film in the magnified image of Figure 2f. Meanwhile, κ_II_ phases seem to be inlaid on the corrosive product film surface without attack.

**Figure 3:**
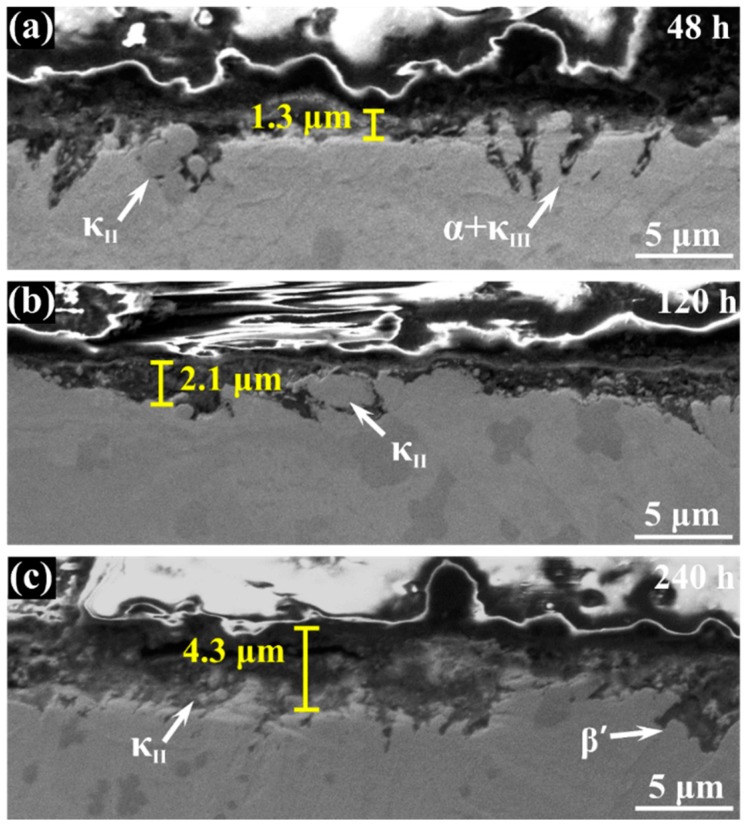
Cross-section morphology of the corrosion product film of NAB alloy after immersion in 3.5 wt % NaCl solution for varying periods of time: (**a**) 48 h; (**b**) 120 h; (**c**) 240 h.

**Figure 4:**
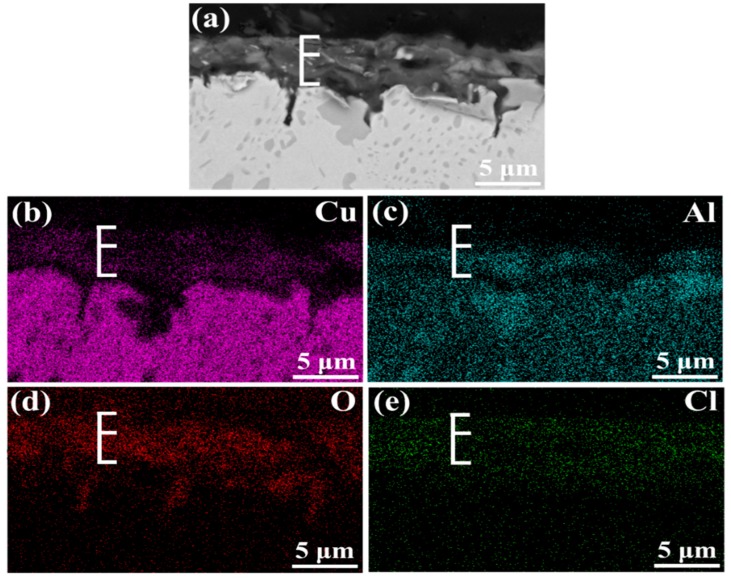
EDS elemental maps of corrosion product film on NAB alloy in cross-section: (**a**) morphology; (**b**) Cu map; (**c**) Al map; (**d**) O map; (**e**) Cl map.

Figure 3 shows the cross-section morphology of the corrosion product film after different immersion times. It is clear that the film becomes thicker with the increase of immersion time, and κ phase particles appear in it. The thickness of corrosive product film reaches 4.3 μm after 240 h immersion while, in first 48 h, it still exists as some bare area which is not covered. The EDS scanning presents the elemental distribution of the film immersed for 240 h, and it indicates that the film of NAB alloy has a duplex structure, as shown in Figure 4. The inner layer is rich in Al and the outer layer is rich in Cu. Some researchers declared that the film is mainly made up of Al_2_O_3_ dense inner layer and Cu_2_O porous outer layer [18,19,20]. It is well accepted that the oxide corrosion products on NAB alloy in sodium chloride solution are generated by the anodic dissolution of metals and the hydrolysis of metallic chlorides, respectively [21,22,23,24,25]:

Cu → Cu^+^ + e^−^, (1)

Cu^+^ + 2Cl^−^ → CuCl_2_^−^, (2)

2CuCl_2_^−^ + H_2_O → Cu_2_O + 4Cl^−^ + 2H^+^, (3)

Al + 4Cl^−^ → AlCl_4_^−^ + 3e^−^, (4)

2AlCl_4_^−^ + 3H_2_O → Al_2_O_3_ + 8Cl^−^ + 6H^+^. (5)

The Al_2_O_3_ layer effectively hampers the corrosive medium and ion transport across the covering film to fresh metal because of the stability and denseness [14,26]. However, the Cu_2_O layer presents weak corrosion protection as in the discontinuity and semiconductor performance [17].

### 3.2. Electrochemical Measurements

**Figure 5:**
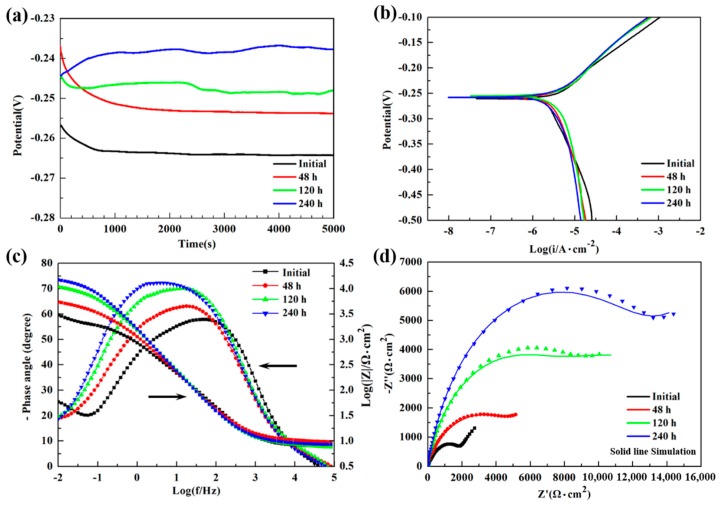
Electrochemical properties of NAB alloy with different immersion time in 3.5 wt % NaCl solution: (**a**) open circuit potential; (**b**) potentiodynamic polarization curves; (**c**) Bode plots with impedance and phase angle; (**d**) Nyquist plots.

Figure 5 shows the changes of electrochemical properties of NAB alloys with different immersion times. The E_OCP_ of each specimen reaches a stable value after a certain period of standing, and the potential shows a positive move with the immersion time increasing (Figure 5a). Figure 5b displays the potentiodynamic polarization curves of NAB specimens. The corresponding E_OCP_, corrosion potential (E_corr_), and corrosion current density (i_corr_) are summarized in Table 2. It is obvious that the value of i_corr_ has the tendency to reduce with the increasing time spent immersed in 3.5 wt % NaCl solution. The specimen immersed for 240 h shows the highest E_OCP_ and lowest i_corr_ in this system, suggesting that it has the best electrochemical corrosion properties compared with others, which could be attributed to the thick and homogeneous corrosive product film. The EIS test is performed by Bode plots and Nyquist plots (Figure 5c,d) to further investigate the corrosion properties of the film after different immersion times. The total impedance modular |Z| at low frequencies is correlated with the barrier layer resistance, which consists of the protective film and the electrical double layer [27]. As the immersion test goes on, the value of |Z| gradually increases, confirming the growth of protective film on the specimen surface, as shown in Figure 5c. The phase maximum angle at intermediate frequencies increases with the immersion time rising (from 57° to 73°), indicating the decrease of corrosion rate and the higher capacitive behavior, which is corresponding to the decrease of i_corr_ [28,29]. In addition, the broadening plateau of phase maximum angle implies that the protective film on the NAB alloy becomes more stable [29,30].

**Figure 6:**
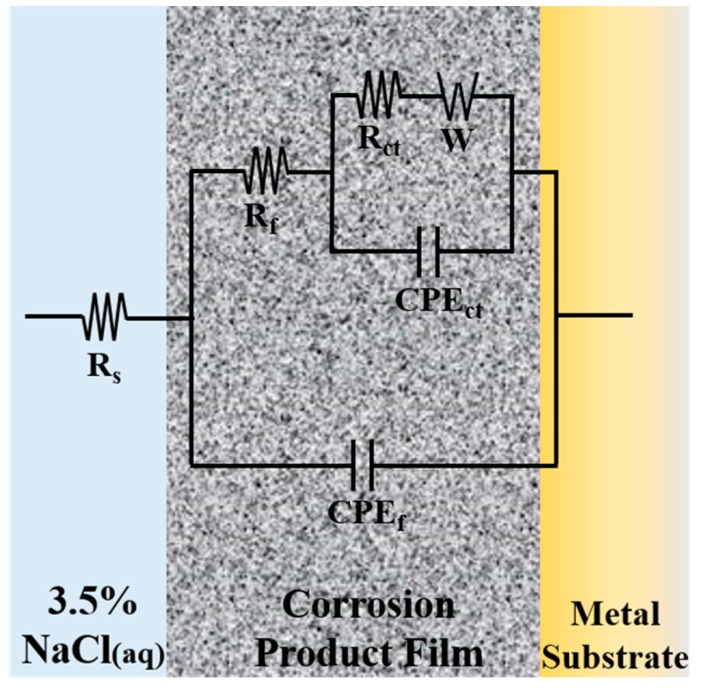
Equivalent circuit model used to fit the Nyquist plots data in Figure 5d.

From the data of Nyquist plots, an equivalent circuit model is designed on Zview software for simulating the corrosion process, as shown in Figure 6. In the model, R_s_ (Ω·cm^2^) is the solution resistance, R_ct_ (Ω·cm^2^) is the charge transfer resistance at the alloy/electrolyte or film/electrolyte interface, CPE_ct_ (μF·cm^−2^) is the non-ideal capacitance of charge transfer, R_f_ (Ω·cm^2^) is the surface film resistance, CPE_f_ (μF·cm^−2^) is the non-ideal capacitance of surface film, and *W* (Ω·s^−1/2^) is the Warburg diffusion element. According to the pattern of the curves, the charge transfer resistance from the high frequency impedance arc region and the surface film resistance from the low frequency impedance arc region could be represented [17]. The simulated results are presented in Figure 5d using solid lines, and the degree of fitting is satisfactory as the standard deviations are in the order of 10^−4^. The related fitted electrochemical parameters are listed in Table 3. The values of R_f_ and R_ct_ dramatically increased over the immersion time, suggesting the improvement of corrosion resistance. This phenomenon is consistent with the reduction of i_corr_ in Table 2. The parameter n_f_, the porosity index of film, increased with immersion time, indicating that the protective film of redeposition was getting thicker and denser, which could be confirmed by morphology observation in Figure 2 and Figure 3. The increase of *W* value and its disappearance at 480 h indicate that the capacity of the film to prevent metals from dissolving to ions increases as the immersion time goes on.

Obviously, the change of electrochemical properties of the specimens is associated with the evolution of protective corrosion product film. Once the surface of fresh NAB alloy contacts the corrosive medium solution, the matrix metals are activated, and the dissolved metal ions diffuse into the solution [31]. The free ions are redeposited on the metal surface by relevant hydrolysis reactions, forming the complex corrosion products [11]. Due to the different compositions and morphologies of various phases, the product films formed on them present different growth rates at early times. The discontinuity and inhomogeneity of films could not effectively protect the metal due to the galvanic cells and the ionic concentration. With the increase of immersion time, the product films are growing on the bare site and getting thicker and more homogeneous, acting as a compact barrier to transport ions and corrosive medium. Therefore, the metallic dissolution and the corrosion reactions are efficiently restrained.

### 3.3. In-Situ SVET Measurements

**Figure 7:**
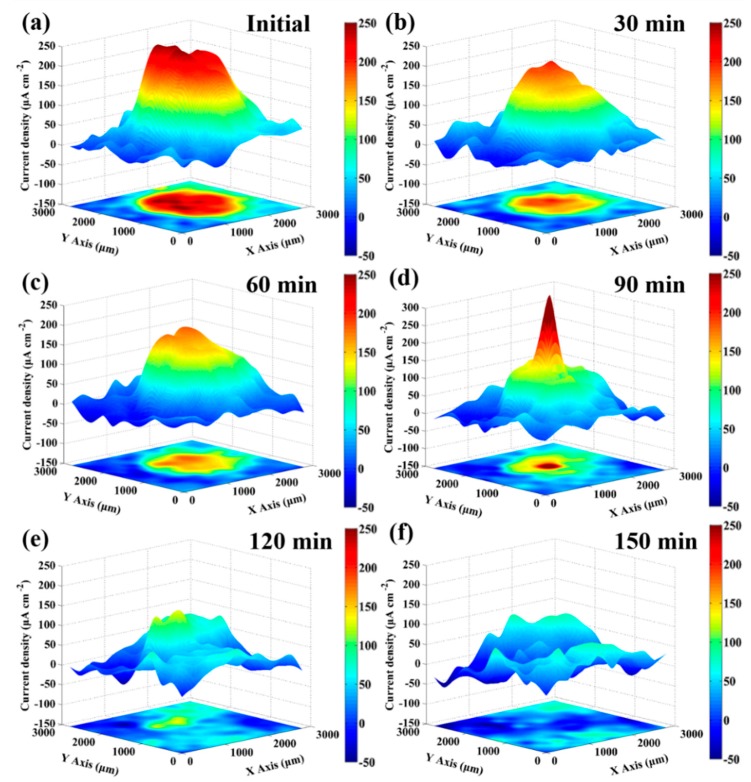
In-situ SVET 3D-maps of the ionic currents measured above the scratch in 3.5 wt % NaCl solution with different times: (**a**) initial; (**b**) 30 min; (**c**) 60 min; (**d**) 90 min; (**e**) 120 min; and (**f**) 150 min.

The conventional electrochemical measurements provide information on the average electrochemical response over the entire sample. To investigate the distribution of local anodic and cathodic activity, and reveal the changes of local electrochemical property, the in-situ SVET measurements were further conducted on a 1.5 mm diameter artificial scratch of bare fresh metal in 3.5 wt % NaCl solution. The evolution of ionic flux signal around the bare fresh metal region was recorded in real time until the signal was approximate to the signal of region covered by the protective film. Figure 7 presents the distribution of current density with in-situ SVET 3D-maps during the period of immersion. At the beginning of the immersion, the current density peak (*i*_A,max_) approaches 260 μA·cm^−2^ in the middle of the circular region, developing a strong anodic activity, shown as a red plateau in Figure 7a. The current density of remaining film surface is around zero, behaving essentially as cathodic. Due to the diffusion of metal cations into solution, a gradation zone appears at the periphery of anodic red region, shown in yellow-green color. At the end of 30-min immersion (in Figure 7b), the *i*_A,max_ on the fresh metal region falls to about 190 μA·cm^−2^, and the anodic red plateau shrinks to the hill, which is observed in the 3D-map. Clearly, the anodic area and the value of anodic current density both decrease dramatically during the first 30-min immersion period. After 30 min of immersion, however, the change of local electrochemical properties follows a different rule.

**Figure 8:**
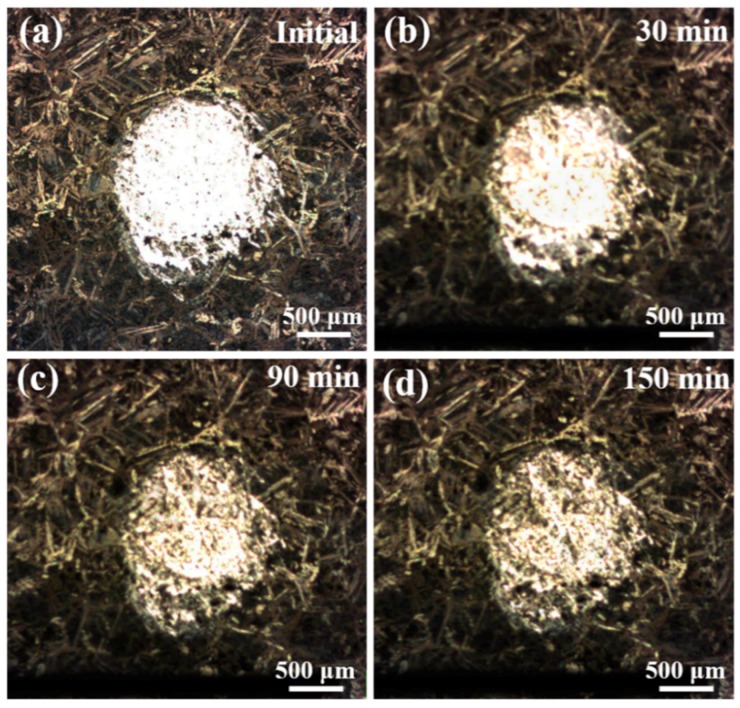
Corresponding optical micrographs of the specimen in the in-situ SVET measurement: (**a**) initial; (**b**) 30 min; (**c**) 90 min; (**d**) 150 min.

Comparing Figure 7c with Figure 7b, from 30 to 60 min of immersion, the *i*_A,max_ gradually reduces to 160 μA·cm^−2^ while the area of anodic region has no obvious change. At the end of 90-min immersion (in Figure 7d), the anodic area shrinks sharply, and it becomes a red spire shape, as shown in the 3D-map. Differing from the previous stages, the peak value of current density increases to 340 μA·cm^−2^ suddenly, instead of decreasing. This phenomenon is related to the significant decline of the ratio between the anode and cathode area. The corrosion product film is constantly deposited and formed at the periphery of red anodic region, and it shrinks the area of anodic activities. The middle of the metal without product film is supposed to transfer the same amount of charge with small area and, thus, the current density shows an incredible rise. After 120-min immersion, the red anodic spire disappeared and only a low yellow anodic hill is detected in Figure 7e. The corrosion product film is deposited and covers on the last small area, resulting in stop of anodic activities. Finally, at the end of 150 min (Figure 7f), the current density of the circular region is approximate to the remaining region with the 240-h immersion protective film, indicating that after 150-min immersion, the protective film on the surface of the NAB alloy has formed again. Figure 8 shows the corresponding optical micrographs of the specimen in the in-situ SVET measurement. With the increase of immersion time, the circular region of bare fresh metal lost its metallic luster and became darker, indicating that the corrosion product gradually covers this region. In addition, it is found that the corrosion product film preferentially formed at the periphery of the fresh metal region.

### 3.4. In-Situ AFM Measurements

**Figure 9:**
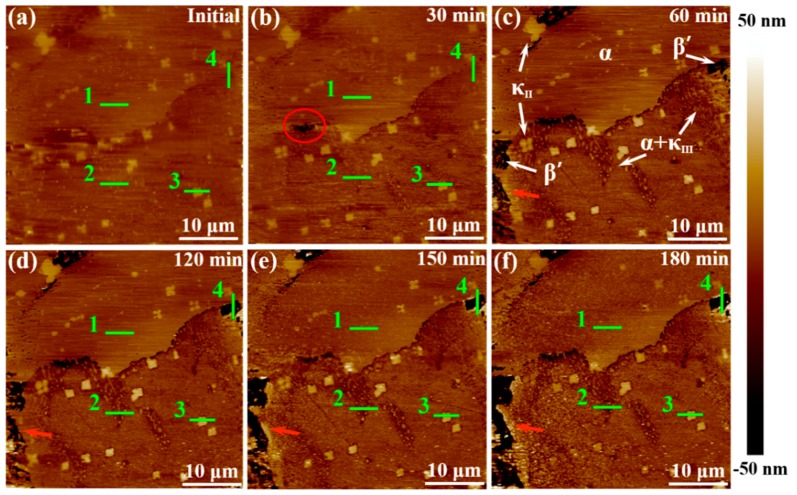
In-situ topography images of NAB specimen surface after exposure to 3.5 wt % NaCl solution for different times: (**a**) initial; (**b**) 30 min; (**c**) 60 min; (**d**) 120 min; (**e**) 150 min; (**f**) 180 min.

**Figure 10:**
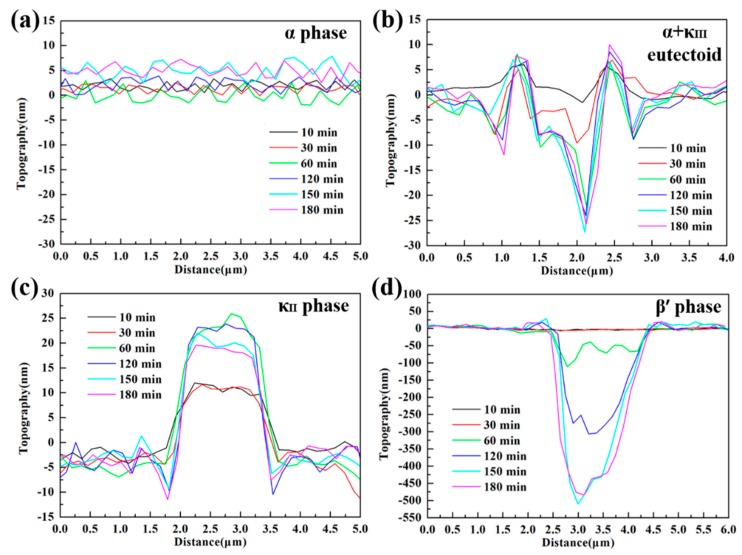
In-situ line profiles corresponding to sites 1, 2, 3, and 4 marked in Figure 9, respectively: (**a**) α phase; (**b**) α + κ_III_ eutectoid structure; (**c**) κ_II_ phase; (**d**) β′ phase.

An in-situ AFM analytical technique with high sensitivity was employed to further quantitatively elucidate the corrosion product film formation process of various phases by using the data of height change, making up for the deficiency of accuracy in SVET measurements. Figure 9 shows the in-situ AFM topography images of NAB specimen surface during 180-min immersion in 3.5 wt % NaCl solution. Before contacting with the corrosive medium, the surface of NAB specimen was vibration polished, and there was no obvious surface relief but only some little κ_II_ bulge, as shown in Figure 9a. After 60 min of immersion, the phases in NAB can be distinguished clearly (in Figure 9c). The brightest areas are κ phases, including κ_II_ and κ_III_. The darkest areas are referred as β′ phases, and the rest of the area largely is α matrix, which could be verified by the distribution feature and their corrosion behaviors [8,32]. The corresponding in-situ line profiles of different phases are shown in Figure 10 (site 1 corresponds to α phase, site 2 corresponds to α + κ_III_ eutectoid structure, and site 3 corresponds to κ_II_ phase, while site 4 corresponds to β′ phase, as marked in Figure 9).

The different phases in NAB alloy present different height changes during the immersion process (in Figure 9), indicating that there is a marked difference between the formations of corrosion product film on various phases. According to Figure 9 and Figure 10, it is clear that the α phases at different locations present different corrosion behaviors. The lamellar α phase within the α + κ_III_ eutectoid structure is corroded by approximately 25 nm at 60 min (in the middle of Figure 10b), whereas the α phase far away from κ phases is not corroded in the whole period, and forms a thin film of approximately 6 nm after 150-min immersion (in Figure 10a). This difference can result from the fact that the α phases in the eutectoid form many microgalvanic cells with lamellar κ_III_ phases, and are dissolved dramatically as anodic parts [33,34]. In addition, since κ_III_ phase is based on aluminum-nickel intermetallic (NiAl), the Ni and Al content of α phase in the α + κ_III_ eutectoid is less than that in the alone α phase. Thus, the decrease of chemical content making the protective film thinner and more unstable also results in this difference [17,35]. After 60 min, the corrosion of the α phases within the eutectoid structure gradually stagnate as a result of protective film formation.

The κ phases, including κ_II_ and κ_III_, present the most stable topography condition during the period of immersion. Neither of the two phases show a deepening corrosion, as shown from the phenomenon that the heights of two phases surfaces have not decreased in Figure 10b,c. The protective film on κ_III_ phases forms rapidly after contacting with the corrosive medium and remains approximately 5 nm, as shown in Figure 10b. The film on the κ_II_ phases, however, starts forming after 30-min immersion, and the maximum thickness can reach 15 nm; it then thins down to stability at 150 min (Figure 10c). The difference of the film formation can be attributable to the difference of morphology and chemical content of two phases. Compared with the particle κ_II_, the lamellar κ_III_ phase has a greater contact area with α matrix, resulting in that electrons exchanging more rapidly and the corrosion product film forming more quickly [3]. On the other hand, κ_II_ phase is based on a Fe_3_Al intermetallic and contains more iron [7]. It is well accepted that the oxides of iron present are fluffier and more unstable compared to the nickel oxides. Therefore, the film on the κ_II_ phase is thicker and, meanwhile, shows solubility, to some extent.

The β′ phases have no obvious corrosion in the first 30 min of immersion, but begin to be corroded quickly towards the end of 60 min, as shown in Figure 9a–c and Figure 10d. The corrosion depth of β′ phases exceeds 50 nm at the end of 60 min. According to earlier works, the β′ phases are found to be vulnerable to corrosion due to the high chemical reactivity of their metastable martensitic phase [6,12,36]. During the next monitoring period, it is found that with the corrosion of β′ phase, the sites around the corroded β′ phases turn lighter (pointed by the red arrows in Figure 9c–f), indicating that the corrosion products prefer to be deposited around the β′ phase region than inside the β′ phases, which is also supported by Figure 10d. From 60 to 150 min of immersion, most of β′ phases maintain a fast corrosion rate until the corrosion depth finally stabilized at around 500 nm. At this time, some corrosion product starts to deposit on β′ phases to form a protective film to inhibit further corrosion of β′ phases. Interestingly, a small piece of β′ phase seems to be corroded earlier, as shown in Figure 9b with a red circle. This phenomenon can be attributed to the small ratio between the anode and cathode area, which causes the more serious and earlier anodic (β′ phase) dissolution reaction in a local galvanic effect.

When the immersion timing is up to 150 min, the corrosion product deposition disperses on almost the whole surface (Figure 9e), and the corrosion depth of each phase is inhibited, as shown in Figure 10. At the end of 180 min (Figure 9f), the corrosion product film of NAB alloy is preliminarily formed, and becomes more uniform.

## 4. Conclusions

In this study, the formation process of corrosion product film on NAB alloy was investigated methodically via morphology characterization, electrochemical measurement, and various in-situ analysis technologies. The corrosion behavior of film during the immersion time and its anti-corrosion mechanisms were demonstrated scientifically. The main analyses are based on the experimental results and reasonable conclusions can be drawn:

1. The corrosion resistance of NAB alloy is due to the protective film covered on the surface, avoiding the contact with corrosion medium and the transportation of ion and charge.
2. Due to difference of location and chemical content, the lamellar α phase within the α + κ_III_ eutectoid is corroded sharply, while the α phase far away from κ phases is well preserved.
3. The κ_II_ and κ_III_ phases present a remarkable corroded resistance as they form a stable and dense protective film within a short time when coming into contact with the corrosion medium. As a result of the metastable martensitic structure and the difficulty to form protective film, the β′ phase suffers the most serious corrosion damage.

## Figures and Tables

**Table 1:** Chemical compositions of each phase in the nickel-aluminum bronze (NAB) alloy (wt %).

Phase	Cu	Al	Fe	Ni	Mn
α	85.4	8.2	2.9	2.4	1.1
β′	84.5	8.6	2.4	3.5	1.0
κ_II_	24.1	18.4	32.8	22.8	1.8
κ_III_	30.6	17.9	19.2	31.2	1.1

**Table 2:** Electrochemical corrosion parameters of specimens after different immersion time in 3.5 wt % NaCl solution.

Immersion Time	E_OCP_ (V)	E_corr_ (V)	i_corr_ (μA/cm^2^)
Initial	−0.264	−0.260	11.35
48 h	−0.253	−0.258	5.32
120 h	−0.248	−0.255	5.04
240 h	−0.237	−0.257	3.78

**Table 3:** Electrochemical equivalent circuit parameters by fitting analysis of specimens after different immersion times in 3.5 wt % NaCl solution.

Immersion Time (h)	R_s_ (Ω·cm^2^)	CPE_f_ (μF·cm^−2^)	n_f_	R_f_ (Ω·cm^2^)	CPE_ct_ (μF·cm^−2^)	n_ct_	R_ct_ (Ω·cm^2^)	W (Ω·s^−1/2^)
0	7.924	163.12	0.6761	1761	121.27	0.8043	317.6	2660
48	9.901	620.98	0.7693	1943	187.72	0.7614	3698	5391
120	8.072	168.15	0.8384	6946	941.58	0.7048	10,362	6242
240	8.640	148.08	0.8712	11,976	1234.8	0.6466	35,939	-
